# Gender Differences in Depression: Assessing Mediational Effects of Overt Behaviors and Environmental Reward through Daily Diary Monitoring

**DOI:** 10.1155/2012/865679

**Published:** 2012-02-16

**Authors:** Marlena M. Ryba, Derek R. Hopko

**Affiliations:** Department of Psychology, The University of Tennessee, Knoxville, 307 Austin Peay Building, Knoxville, TN 37996-0900, USA

## Abstract

Gender differences in the prevalence of depression are well documented. To further explore the relation between gender and depression, this study used daily diaries to examine gender differences within thirteen behavioral domains and whether differential frequency of overt behaviors and environmental reward mediated the relationship between gender and depression severity. The sample included 82 undergraduate students [66% females; 84% Caucasian; Mean age = 20.2 years]. Overall, females engaged in a significantly greater breadth of behavioral domains and reported a higher level of environmental reward. Females spent more time in the domains of health/hygiene, spiritual activities, and eating with others. Males spent more time in the domains of physical activity, sexual activity, and hobbies and recreational experiences. Females found social activities, passive/sedentary behaviors, eating with others, and engagement in “other” activities more rewarding. Gender had a significant direct effect on depression severity, with females reporting increased depression. This effect was attenuated by the mediator (total environmental reward) such that to the extent females exhibited increased environmental reward, the gender effect on depression was attenuated. These data support behavioral models of depression, indicate increased reinforcement sensitivity among females, and have clinical relevance in the context of assessment and behavioral activation interventions for depression.

## 1. Introduction

Gender differences are apparent in prevalence rates of certain mental health problems. For example, females are more likely to present with internalizing disorders such as depression and anxiety [1–4], whereas men have a higher prevalence of several externalizing disorders, including antisocial personality disorder and substance abuse [5–7]. Pertaining to gender differences in depression, beginning in late adolescence [8], and continuing through adulthood, it is widely established that depression is more common among females (21%) than males (13%; [9]). Many factors may account for this gender difference, including *biological* influences such as genetics, hormones, adrenal functioning, and neurotransmitter systems, as well as *psychosocial* variables such as more frequent victimization and trauma in childhood, gender role factors (e.g., competing social roles, role restrictions), interpersonal orientation such as increased vulnerability to the emotional pain of others, being more prone to rumination, differential attributional styles, and greater reactivity to stress in terms of biological responses, self-concept, and coping styles [4, 10–19]. Anxiety disorders are highly coexistent with depression, are more prevalent among females, and also may contribute to the onset, maintenance, and severity of depressive episodes [2, 20–22]. 

Behavioral theories explain the development and persistence of depression as the result of decreased response-contingent positive reinforcement (RCPR) [23–27]. A low rate of RCPR is proposed as the critical mediator between overt behaviors and depression severity [26, 28], RCPR defined as an increase in the frequency or duration of behavior as a result of positive or pleasurable outcomes. Minimal environmental (and social) reinforcement results in the extinction of “healthy” behaviors and consequently the dysphoria and passivity that often characterize depression. A low rate of RCPR is a product of decreased availability of potential reinforcers in the environment, inabilities to experience rewarding contingencies due to inadequate instrumental behaviors such as social, occupational, or academic skills, and increased exposure to distressing or unpleasant events [26, 29]. Supporting behavior theory, several studies highlight relationships between pleasant events and mood state, with individuals reporting fewer positive events, decreased environmental reward, and more limited abilities to obtain reinforcement endorsing greater depression [28, 30–34]. Depressed individuals also tend to engage in fewer interpersonal behaviors, suggesting that insufficient social interaction and decreased social reinforcement may predict negative affect [35–37].

Also supporting behavioral models of depression, behavioral activation interventions that focus on increasing RCPR are largely effective, with meta-analyses supporting their efficacy such that behavioral activation is now considered an empirically validated treatment for depression [38–41]. In one of the more compelling studies, behavioral activation was comparable to antidepressant medication and superior to cognitive therapy in treating severe depression [42], results that were maintained at 2-year follow-up [43]. Behavioral activation also has been effectively used with depressed patients in a variety of settings and among samples with divergent medical and psychiatric problems [44–52].

Considering the well-documented gender differences in depression and strong empirical support for behavioral models of depression, there is a pressing need to explore potential gender differences across a breadth of behavioral domains and determine whether these differences contribute to increased depression in females. Indeed, depressed and nondepressed individuals have been shown to differ substantially in terms of overt behavior. For example, in addition to increased social avoidance alluded to earlier, depressed individuals generally report participating in fewer rewarding and pleasurable activities [30, 33, 34] and engage in fewer physical and educational behaviors [31, 53]. Depressed individuals also generally exhibit a slower and more monotonous rate of speech, take longer to respond to the verbal behavior of others, exhibit an increased frequency of self-focused negative remarks, and use fewer “achievement” and “power” words in their speech [37, 54]. Depressed and nondepressed individuals also differ in their non-verbal behavior. Depressed individuals smile less frequently, make less eye contact, more frequently hold their head in a downward position, and are rated as less competent in social situations [54–56]. Accordingly, understanding gender differences in overt behavioral patterns may allow further insight into the higher prevalence of depression in females and potentially have important assessment and treatment implications. If males and females differ in the frequency and possibly reward derived from certain overt behaviors, it is conceivable that these differences could contribute to the development and maintenance of psychological problems such as depression [26]. In such cases, it would be feasible to proactively recommend healthy behavioral repertoires and modify treatment interventions to more adequately address psychological distress while taking gender into account. As an important step in this process, it is necessary to more validly assess potential gender differences in overt behaviors in the context of major life domains [57].

The primary aim of this study was to evaluate differences between males and females in activities assessed via self-monitoring through daily diaries. Relative to self-report strategies that retrospectively assess overt behaviors, a more ecologically valid method of determining the frequency of behaviors may be through use of such daily diaries [30]. Studies incorporating daily diaries have found daily ratings of behaviors and depression symptoms to correlate strongly with self-report and clinician-rated measures of depression [30, 31, 58–60]. Similar daily diary designs have demonstrated adequate internal consistency and good convergent and discriminate validity in research on anxiety [61, 62] and other symptom presentations [63–67]. Using this methodology as a novel approach to exploring behavioral gender differences, it was hypothesized that females would engage in more passive and sedentary behaviors, while males would engage in more physical and active behaviors as evolutionary theory and social learning models would suggest [68]. Second, in addition to increased behavioral frequency and based on matching theory [69], it was hypothesized that males and females would find these specific activities more rewarding. Finally, based on behavioral models of depression [24, 26], it was hypothesized that decreased engagement in nondepressive healthy behaviors and diminished environmental reward would significantly mediate the relationship between gender and depression severity [70].

## 2. Method

### 2.1. Participants

Participants included 82 undergraduate students (females: *n* = 54; males: *n* = 28) from an introductory psychology class at a large southeastern university. The sample consisted of 69 Caucasians (84.1%), 8 African Americans (8.5%), and 6 (7.3%) participants who self-identified as Asian American. The mean age of participants was 20.2 years (SD = 3.9 years). All participants received course-related research credit for their participation and the research was approved by the University of Tennessee Institutional Review Board.

### 2.2. Assessment Measures

Participants completed the Beck Depression Inventory-II (BDI-II; [71]), a 21-item measure of depression symptom severity, each of which is rated on a 4-point Likert scale (0–3 point anchors), with items summed to form a total score. The instrument has excellent internal consistency (*α* = .92) as well as strong convergent validity with other measures of depression [71, 72]. Internal consistency in this sample was excellent (*α* = .93). For the current sample (BDI-II: *M* = 11.7, SD = 7.8), females reported increased depressive symptoms (*M* = 13.0, SD = 8.0) relative to male participants (*M* = 9.3, SD = 7.1) (*t*  (80) = 2.11, *P* < 0.05).

### 2.3. Procedure

Participants met with an experimenter on two occasions. During the first meeting, participants first completed the BDI-II and a demographic form. Participants were then given a packet that included seven daily activity-monitoring forms and detailed instructions. Participants were instructed to record all of their behaviors and activities for the following week. These daily forms contained space for participants to record their behavioral data from 8 A.M to 2 A.M, within half-hour intervals. Participants were also encouraged to be as honest as possible and to record their behaviors every couple of hours to help them accurately recall their behaviors. They were then asked to code each behavioral activity according to *one* of the following categories:

1. social: time with friends, family, boyfriend or girlfriend, and so forth;
2. physical: hiking, biking, walking to class, any other exercise, and so forth;
3. health/hygiene: showering, bathing, brushing teeth, being at the doctor or dentist, and so forth;
4. spiritual: attending church, engaging in prayer/ meditation, reading religious text, and so forth;
5. educational: classes, homework, lectures, computer work, and so forth;
6. passivity/sedentary: napping, sitting, watching television, Internet surfing for fun, and so forth;
7. sexual: intimate physical acts, intercourse, masturbation, and so forth;
8. employment/volunteering: working at your job, babysitting, helping the elderly, and so forth;
9. hobbies and recreation: reading, drawing, writing, scrapbooking, playing music, and so forth;
10. eating alone: snacking, meals, and so forth;
11. eating with others: snacking, meals, and so forth;
12. travel: commuting to school, home, work, flying, traveling to foreign countries, and so forth;
13. other: any behavior not coded in domains 1–12.

Additionally, participants were instructed to engage in their normal routines and to not alter their behaviors for the purpose of this study. For each behavior listed on their daily activity-monitoring forms, participants indicated the degree to which they found the activity to be rewarding (on a 1 (minimally rewarding) to 4 (highly rewarding) Likert scale). Finally, participants were provided with an explanation as to what constituted overt behavior and were asked *not *to record specific thoughts, physiological responses, feelings, and emotional experiences. Participants returned approximately 1 week later (pending participant and experimenter availability), returned their daily diaries, and completed a postassessment BDI-II.

## 3. Results

The total duration of time (hours per week) spent in each activity domain was calculated for each participant and is presented in Table 1. For the entire sample (*n* = 82), the most commonly reported behaviors were as follows, presented in descending order based on the percentage of time activities engaged in during the monitoring week: educational (26%), passivity/sedentary (25%), social (13%), eating with others (6%), employment/volunteering (6%), travel (5%), health/hygiene (4%), hobbies and recreation (4%), physical (3%), other (3%), eating alone (3%), spiritual (1%), and sexual (1%). independent-sample *t*-tests were used to examine whether the mean duration of time in each activity domain statistically differed as a function of gender. Estimated Cohen's *d* [73] is presented as a measure of effect size (*d* = 0.20 = small; *d* = 0.50 = medium; *d* = 0.80 = large). As indicated in Table 1, on a more global level, females engaged in a significantly greater number of behavioral domains and reported a higher level of overall environmental reward relative to males. On a more specific level of analysis, females reported spending a greater duration of time in the behavioral domains of health/hygiene, spiritual activities, and eating with other individuals. In contrast, males reported spending more time in the behavioral domains of physical activity, sexual activity, and hobbies and recreational experiences. Males and females did not differ in the duration of time spent in the following domains: social, educational, passive/sedentary, employment, travel, time spent eating alone, or engagement in “other” activities. Also presented in Table 1, the average reward value recorded on the daily diaries for each behavioral domain was compared as a function of gender. In relation to males, females found social activities, passive/sedentary behaviors, eating with others, and engagement in “other” activities more rewarding. There were no group differences in reward ratings in the behavioral domains of eating alone, physical activity, health/hygiene, spiritual, educational, sexual, employment, recreation/hobbies, or travel activities.

### 3.1. Mediation Analyses

Mediation analyses (e.g., tests of indirect effects) were conducted using a bootstrapping method [74], which has a lower Type II error rate and greater statistical power than the traditionally used causal steps approach advocated by Baron and Kenny [75] (see [74, 76–78]). Bootstrapping techniques were performed in line with recommendations by Preacher and Hayes [74], with *k* = 5,000 re-samples and 95% bias-corrected and accelerated (BCa) confidence intervals (CIs) used to evaluate indirect effects. BCa confidence intervals include corrections for median bias and skew [79]. The use of 95% confidence intervals is equivalent to testing for significance at the 0.05 level. The confidence interval estimates are reflective of the 5000 resamples and the point estimates indicate best estimations of single sample population parameters. Mediation was considered to have occurred if the 95% BCa confidence intervals generated by the bootstrapping method did not contain zero. Mediation analyses were conducted only for those behavioral domains and reward values that were identified as differing as a function of gender. For all mediation analyses, gender was the independent variable and depression severity (BDI-II) was the dependent variable. Consistent with prior studies [30, 31, 70] depression severity was based on the average BDI-II score from both administrations. This strategy was used to obtain a more accurate index of psychological functioning during the one-week assessment period as opposed to using either the time 1 or time 2 administration. As presented in Table 2, daily diary-measured total overall reward significantly mediated the relationship between gender and depression severity. In terms of other diary-based variables identified as differing as a function of gender, time spent in hobbies and recreational activities and reward value of “other” activities also mediated the relationship between gender and depression severity.

## 4. Discussion

In the last several decades, substantial research has explored gender differences on a wide range of abilities and behaviors and the potential implications of these differences on a number of outcome variables, including but not limited to academic performance, occupational status, and mental health functioning. The current investigation expanded on these initiatives by utilizing a daily diary monitoring methodology to examine gender differences on thirteen primary life domains that are considered fairly comprehensive insofar as capturing major categories of overt human behaviors [57]. In contrast to past research, behavioral gender differences were identified using a more direct and naturalistic assessment method [80] that extended beyond retrospective behavioral accounts, minimized experimental demand characteristics, and did not rely on experimental manipulations to infer relationships between variables. The study also was novel in the aim of addressing how gender differences in overt behavior might mediate the well-established relation between female gender and increased depression prevalence [1, 9]. Consistent with evolutionary and social learning theories of behavioral gender differences [68], results supported the notion that males and females differ in the duration of time engaged in particular behavioral domains as well as reward experienced in different domains. As predicted, males engaged in more active behaviors for significantly longer time durations, including physical-, sexual-, and recreational-based activities. In contrast, females spent more time engaged in social activities such as spiritual and religious behaviors as well as dining with others. As indicated by increased duration of time in health- and hygiene-based activities, females also generally appeared more concerned with physical appearance. Also consistent with hypotheses, females reported social behaviors (including eating with others) as well as passive and sedentary activities to be more rewarding. 

Contrary to the matching theory hypothesis [69], all high-frequency behaviors were not necessarily endorsed as more rewarding. Furthermore, males did not report greater derived reward in any behavioral domain relative to females. One explanation for these findings involves possible gender differences in terms of reactivity to self monitoring [81, 82]. Second, it is conceivable that the perceived level of reward derived from engaging in particular behaviors is less operational for males than females, with the former gender potentially requiring less salient or potent reinforcement schedules to maintain overt behaviors. Third, the findings of this paper support previous research indicating that females are more communal in nature [83]. For example, it was found that women spent more time eating with others and engaging in health/hygiene and spiritual behaviors. While eating with others is clearly a communal activity, it is feasible that health/hygiene and spiritual behaviors serve the function of increasing the likelihood of rewarding communal activities. The finding that females also reported significantly greater reward associated with social activities also supports this assumption. 

Interestingly, collapsed across all behavioral domains, females reported increased overall reward associated with overt behaviors as well as participation in a significantly greater breadth of behavioral domains. Intriguingly, and contrary to behavioral theory and research supporting the link between increased environmental reward and reduced depressive affect [24, 26, 30, 70, 84], females also reported increased depression severity on the BDI-II. To address this apparent anomaly, reference to mediational analyses is necessary. Specifically, although gender had a direct effect on depression severity, this effect was attenuated by the mediator (total environmental reward) such that to the extent that females exhibited increased self-reported environmental reward, the gender effect on depression was reduced. In other words, when you control for the significant relation between the mediator (environmental reward) and depression, gender and depression are less related—females are more likely to report elevated depression only when environmental reward also is lower. Thus, increased environmental reward serves to buffer the association between gender and depression such that when environmental reward is a statistical covariate, gender no longer is significantly associated with depression in this sample. This finding is entirely consistent with behavioral models of depression and supports conceptual foundations of behavioral activation treatment interventions designed to increase exposure to environmental reward and response-contingent positive reinforcement [38, 39, 41]. Moreover, these data suggest that at least one plausible mechanism to address gender differences in depression may be through concerted efforts to increase environmental reward and reinforcement in depressed females. Indeed, in a recently conducted randomized controlled trial examining the efficacy of behavioral activation for depressed women with breast cancer, the intervention reduced depression significantly and was associated with strong effect sizes, and treatment gains were maintained through 12-month follow-up [47]. Also noteworthy, the significant mediational effect of hobbies and recreational activities suggests that increasing the frequency of these behaviors may potentially attenuate depressive symptoms. Whether specifically targeting this behavioral domain among females with increased depression severity would be an effective behavioral intervention is an empirical question worthy of investigation. Indeed, it has recently been demonstrated that a behavioral activation protocol focused exclusively on religious behaviors effectively reduced depression [85].

Although study findings are highly provocative, several limitations are noteworthy. First, behavioral contingencies are experienced on a continuous basis. Accordingly, although perhaps an advancement, even the present methodology of monitoring activities in half-hour intervals does not allow measurement of the entire spectrum of overt behaviors and operant relations. Second, functional qualities of behaviors and the frequency of punished behaviors as a function of gender were not explored in the current study [26, 86]. This limitation is significant given the importance of functional relationships and environmental suppressors in conceptualizing the development and persistence of depression [87]. Third, although participants reported compliance with monitoring procedures when queried postexperimentally, we cannot be certain as to whether diaries were completed at reliable and regular intervals. Indeed, this limitation is inherent in a majority of studies that incorporate diary methods. Future studies can increase participant compliance with the use of Internet-based assessment or palm pilots [88]. Fourth, it is possible that unmeasured variables may account for unique variance in behaviors that in the present study were attributable to gender differences. For example, controlling for a masculine versus feminine gender identity (perhaps using the MMPI-2) would help determine the incremental validity of gender as a predictor of frequency and reward value of social behavior. Fifth, reward ratings and their association with negative affect were not assessed as a function of temporal factors. Accordingly, although a behavior may initially be perceived as rewarding, delayed negative consequences might occur that could subsequently affect self-reported reward and negatively impact mood. Longitudinal work is necessary to address this issue. Sixth, attention to private behaviors was not undertaken in this study, and therefore the presence of potential gender differences in covert behaviors cannot be addressed. Finally, some measurement error might have been associated with behavioral coding strategies. As the study required participants to code their activities, and although they received instruction on this process, they did not receive extensive guidance or training, which may have resulted in problems with inter-rater reliability and decreased study power. Related to this limitation, results based on daily self-ratings of environmental reward could have been strengthened (i.e., convergent validity) by including a psychometrically sound self-report measure of this construct such as that used in prior studies [23].

In closing, study findings indicate gender differences in depression severity as well as the frequency and reward value of certain overt behaviors. Most substantially, consistent with behavioral theories of depression, mediation analyses indicated that one potential reason for gender differences may be that level of environmental reward may be more consequential toward eliciting depressive affect in females relative to males. Perhaps the most parsimonious explanation for this finding, albeit in need of replication, is the notion that females may have increased reinforcement sensitivity or reward responsiveness [89–91]—that is, behavioral activation systems more functional in attempting to seek rewards, such as a predilection towards novel experiences, spontaneous behavior, and exciting activities [92, 93]. This finding is highly unique and contributes to the multidimensional perspective of gender differences in depression. As this study was conducted with a nonclinical sample, it is conceivable to predict a magnification of already large effect sizes with a well-diagnosed clinical sample of depressed adults. Replication of study findings would provide additional support and utility for behavioral assessment and activation interventions among depressed individuals, in particular females. Further systematic research in this area will be critical toward continued refinement of behavioral interventions and conceptualizing the role of gender differences as they pertain to emotional health problems such as depression.

## Figures and Tables

**Table 1:** Time duration and reward value of overt behaviors as a function of gender.

Behavioral domain	Male	Female	*t*	*P*	*d*
Total domains engaged	9.5 (1.5)	10.1 (1.3)	2.04	<0.05	0.59
Total average reward	2.6 (0.6)	2.9 (0.4)	2.13	<0.05	
Social	28.5 (24.6)	35.4 (16.4)	1.51	0.13	
Social reward	3.2 (0.6)	3.6 (0.3)	3.20	<0.01	0.84
Physical	12.2 (15.8)	6.8 (7.7)	2.04	<0.05	0.43
Physical reward	2.9 (1.0)	3.0 (0.8)	0.14	0.89	
Health/hygiene	6.2 (5.4)	11.5 (5.6)	4.10	<0.01	0.96
Health/hygiene reward	2.6 (0.8)	2.6 (0.7)	0.19	0.85	
Spiritual	1.3 (3.0)	4.3 (6.6)	2.30	<0.05	0.59
Spiritual reward	2.9 (0.9)	3.5 (0.8)	1.48	0.15	
Educational	62.7 (22.0)	67.6 (21.1)	0.98	0.33	
Educational reward	1.8 (0.6)	1.9 (0.6)	0.65	0.52	
Passivity/sedentary	68.0 (21.9)	61.8 (20.0)	1.30	0.20	
Passivity/sedentary reward	3.1 (0.7)	3.4 (0.6)	2.00	<0.05	0.46
Sexual	2.3 (4.7)	0.8 (2.3)	2.02	<0.05	0.41
Sexual reward	3.7 (0.6)	3.7 (0.4)	0.15	0.88	
Employment	16.6 (25.0)	14.0 (18.9)	0.53	0.60	
Employment reward	2.4 (0.8)	2.7 (0.8)	0.89	0.38	
Hobbies/recreation	15.6 (26.0)	6.3 (8.5)	2.40	<0.05	0.51
Hobbies/recreation reward	3.2 (0.6)	3.4 (0.5)	1.23	0.23	
Eating alone	6.8 (4.7)	6.2 (6.3)	0.44	0.66	
Eating alone reward	2.7 (0.7)	2.7 (0.8)	0.19	0.85	
Eating with others	11.9 (8.5)	16.4 (10.0)	2.04	<0.05	0.48
Eating with others reward	3.1 (0.7)	3.5 (0.5)	2.81	<0.01	0.66
Travel	13.8 (11.8)	11.9 (12.9)	0.65	0.52	
Travel reward	2.0 (0.7)	2.1 (0.8)	0.11	0.91	
Other	5.4 (7.7)	8.8 (8.4)	1.77	0.08	
Other reward	1.8 (0.7)	2.4 (0.8)	2.82	<0.01	0.80

(1) For behavioral domains, time is presented in hours per week. (2) “*d*” refers to Cohen's effect size [73].

**Table 2:** Indirect effects of gender on depression through duration and reward values of overt behaviors using bootstrapping technique (*N* = 82: 5000 bootstrap samples).

	Point estimate	BCa 95% CI	
		Lower	Upper
*Simple mediation*			
Total domains engaged	−.29	−1.90	0.38
Total average reward	−.82	−2.90	−0.02*
Social reward	−1.20	−2.43	0.01
Physcial duration	.11	−0.42	1.07
Health/hygiene duration	−1.07	−3.29	0.43
Spiritual duration	−.47	−1.38	0.47
Passive/sedentary reward	−1.12	−2.42	0.04
Sexual duration	−.17	−0.99	0.47
Hobbies/recreation duration	−1.56	−5.66	−0.14*
Eating with others duration	−.68	−1.96	0.02
Eating with others reward	−1.13	−3.15	0.96
“Other behaviors” reward	−1.72	−4.57	−0.72*

BCa CI = Bias-corrected and accelerated confidence interval. Confidence intervals containing zero are considered nonsignificant.*P < 0.05.
